# Nutrient transporter pattern in CD56^dim^ NK cells: CD16 (FcγRIIIA)-dependent modulation and association with memory NK cell functional profile

**DOI:** 10.3389/fimmu.2024.1477776

**Published:** 2024-11-13

**Authors:** Davide De Federicis, Cristina Capuano, Daniel Ciuti, Rosa Molfetta, Ricciarda Galandrini, Gabriella Palmieri

**Affiliations:** ^1^ Department of Experimental Medicine, Sapienza University of Rome, Rome, Italy; ^2^ Department of Molecular Medicine, Sapienza University of Rome, Rome, Italy; ^3^ Departmental Faculty of Medicine and Surgery, UniCamillus-Saint Camillus International University of Health and Medical Sciences, Rome, Italy

**Keywords:** memory natural killer cells, CD16, nutrient transporters, IFN-γ, mTORC1

## Abstract

**Background:**

Human memory NK cells represent a heterogeneous CD56^dim^ population that expands and persists in human cytomegalovirus (HCMV)-seropositive healthy individuals. They are characterized by the preferential, not fully overlapping, expression of NKG2C (activating receptor for HLA-E) and CD57 maturation marker, and by the lack of FcεRIγ adaptor chain. Hyperresponsiveness to Fcγ receptor IIIA (CD16) engagement represents the distinctive functional signature of memory NK cells. Although CD16 engagement was shown to acutely enhance glycolytic and oxidative pathways, its capability to induce a persisting metabolic reprogramming of human NK cells is poorly understood yet.

**Results:**

Here, we describe the peculiar nutrient transporter expression pattern of FcεRIγ^-^ memory NK cells, characterized by higher levels of CD98 neutral amino acid antiporter and CD71 transferrin receptor, and lower expression of GLUT1 glucose transporter, with respect to FcεRIγ^+^ conventional NK cells. Although CD16 engagement acutely enhances glycolytic and oxidative pathways, its capability to induce a persisting metabolic reprogramming of human NK cells is poorly understood yet. Our results firstly show that sustained CD16 engagement by contact with IgG-opsonized target cells induces the mTORC1-dependent upregulation of CD98 and CD71 nutrient receptors on CD56^dim^ NK cells, in a transporter-specific fashion, that is finely tuned by cell-dependent (grade of functional maturation, and memory or conventional lineage) and stimulus-dependent (time length and cooperation with cytokines) factors. We also demonstrate that CD98 antiporter function is required for CD16-dependent IFN-γ production, and that enhanced CD98-mediated neutral amino acid uptake associates with heightened memory NK cell functional response.

**Conclusion:**

Collectively, our work documents that CD16 engagement leads to a metabolic rewiring of human NK cells and suggests that a distinct nutrient transporter expression pattern may contribute to memory NK cell peculiar functional features.

## 1 Introduction

Human memory, or adaptive, NK cells represent a highly heterogeneous population of mature CD56^dim^ NK cells, stably and variably expanded in human cytomegalovirus (HCMV)-seropositive healthy individuals (1–6). Distinctive features of the memory pool are the long persistence *in vivo* (3, 7, 8), the further and transient expansion following coinfection with selected pathogens (2, 5, 9), an epigenetic and metabolic profile similar to that of CD8^+^ memory T cells (7, 8, 10–13), and marked hyperresponsiveness to CD16 low-affinity IgG Fc receptor (FcγRIIIA) (1–8, 14–21). CD16 represents the most powerful activating receptor on human mature CD56^dim^ NK cells; its engagement by contact with IgG-opsonized cells or immune complexes unleashes the whole spectrum of effector functions, i.e. cytotoxic activity, cytokine and chemokine production, and may also trigger proliferation, regulate survival, and promote functional priming, under specific conditions (22–27).

HCMV-dependent memory NK cells are variably identified by the preferential, although not completely overlapping, expression of a combination of surface receptors that bear functional implications; they include CD94/NKG2C (activating receptor for the non-classical MHC class I molecule HLA-E), CD57, that marks highly differentiated cells with low proliferative potential and high functional competence (21, 28–30), and self specific-skewed KIR repertoire (20). The complex epigenetic and transcriptional remodelling supports the enhanced capability to produce IFN-γ, along with the reduced expression of selected intracellular signalling intermediates, such as SYK tyrosine kinase, FcϵRγ and EAT-2 cytoplasmic adaptors, and PLZF transcription factor (1, 4, 7, 8, 10, 11, 13, 31). In particular, the absence of immunoreceptor tyrosine-based activating motif (ITAM)-bearing Fcε Receptor I γ (FcεRIγ) adaptor, by shifting the equilibrium towards the exclusive association of CD16 with TCRζ homodimers, was mechanistically linked to memory cell hyperresponsiveness to receptor engagement, despite reduced surface expression levels (14, 17, 32).

Intracellular metabolic pathways provide the necessary amounts of energy and biosynthetic precursors to enable specialized functions; to this end, the regulated acquisition of nutrients, such as glucose, iron and amino acids from the extracellular environment, is mediated by specialized transmembrane transporters (33, 34). The solute-linked carrier (SLC) 7A5, associated with CD98 heavy chain (also called SLC3A2), represents the exclusive antiporter for the intake of large neutral amino acids (LNAAs), such as leucine, isoleucine, and valine, in exchange for intracellular glutamine, in NK cells (35, 36). Transferrin receptor protein 1 (TfR1) or CD71 internalizes iron-bound transferrin, to promote the entrance of iron, a critical cofactor of many enzymes involved in DNA synthesis or repair, oxygen transfer and mitochondrial function, among others; its surface levels typically correlate with the rate of cell proliferation (37, 38).

mTORC1 is a critical signalling node that links sensing of nutrients, such as amino acids, glucose and oxygen, to the coordinated regulation of cellular metabolism; mTORC1 signalling inhibits autophagy and stimulates mRNA translation, glycolysis, lipid and nucleotide synthesis, thus supporting ATP production and the synthesis of the major macromolecules required for cell growth (39, 40). CD98/SLC7A5-mediated entrance of neutral amino acids, in particular leucine, in exchange for glutamine, is thought to be essential to sustain basal activation of mTORC1 and allow its further stimulation by growth factors (39, 41–43). On the other hand, mTOR activity regulates the expression of the antiporter in a feed-forward process (44).

At steady state, NK cells display low rates of glycolysis and oxidative phosphorylation. The enhancement of both pathways, together with mitochondria fitness, represent a robust metabolic response that enables the survival and effector functions of activated NK cells (33, 34, 43–48). Several studies have characterized the capability of cytokines such as IL-2, IL-12, IL-15, IL-18, to alter basal NK cell metabolic profile, promote mTOR activation, and upregulate the expression of CD98, CD71 and GLUT1 nutrient transporters. This metabolic reprogramming was required, to a variable extent, for cytokine-induced cytotoxic competence, cytokine production and proliferation, and for *in vivo* NK cell expansion and antiviral protection (34, 46, 49, 50). On the other hand, few studies have addressed the possible metabolic rewiring promoted by activating receptors that mediate target cell recognition (51–53). In particular, receptor-triggered acute upregulation of glycolysis, oxidative phosphorylation and mTOR activity was required for optimal CD16-dependent IFN-γ production and NK cell priming (19, 54, 55), but either the upstream events that enable this metabolic switch and its possible persistence are completely unknown.

Several metabolic peculiarities have been previously noted in the heterogeneous population of HCMV-expanded memory NK cells, and may be mechanistically linked to their enhanced survival and hyperresponsiveness to CD16 engagement (45, 50, 56, 57). CD56^dim^FcεRIγ^-^ displayed higher basal levels of Bcl-2 (15, 16, 21), while the NKG2C^+^CD57^+^ partially overlapping subset showed enhanced basal glycolytic and oxidative activity with respect to NKG2C^-^CD57^+^ counterpart (12). Acute CD16 stimulation led to enhanced glucose uptake in IL-15-stimulated NKG2C^+^CD57^+^ cells (12), and to amplified activation of the mTOR pathway in CD56^dim^FcεRIγ^-^ population (19). However, the expression pattern of nutrient transporters on memory NK cells and the possible impact of CD16 engagement remain still unexplored.

Here, we provide evidence that FcεRIγ^-^ memory NK cells express a distinct profile of nutrient receptors, characterized by increased CD98 and CD71 and lower GLUT1 levels, with respect to FcεRIγ^+^ conventional NK cells. Persistent CD16 triggering induced the upregulation of CD98 and CD71 on both memory and conventional NK cells, with different kinetics, in a mTORC1-dependent manner. Furthermore, CD98-dependent neutral amino acid uptake sustained CD16-triggered IFN-γ production of memory and conventional NK cells via either mTORC1-dependent and -independent pathways, and the enhanced CD98-dependent transporter function positively associated with the heightened CD16-triggered IFN-γ secretion of memory NK cells.

## 2 Materials and methods

### 2.1 Donor cohort recruitment

Healthy donors were recruited by the Blood Transfusion Centre of Policlinico Umberto I in anonymized form and gave written informed consent. All the procedures involving participants were in accordance with the declaration of Helsinki. The study was approved by the Ethics Committee of Sapienza University of Rome (approval number 639/16 RIF/CE 4179). Only donors with a percentage of CD56^dim^FcεRIγ^−^ NK cells≥5%, evaluated by flow cytometry, were included in this study.

### 2.2 Peripheral blood mononuclear cell isolation

PBMC were obtained by lymphoprep (Ficoll-Hypaque, Cedarlane, Burlington, ON, Canada) density gradient centrifugation of heparinized peripheral blood samples. Then, PBMC were washed with phosphate buffered saline (PBS) and used for *in vitro* assays, immunostaining and cytofluorimetric analysis.

### 2.3 Cell line

CD20^+^ lymphoblastoid Raji B cell line, provided by Dr. F. D. Batista (The Ragon Institute of Mass General, MIT and Harvard, Cambridge, MA, USA) was cultured in RPMI 1640 medium (Euroclone, Italy), supplemented with 10% heat-inactivated Fetal Calf Serum (FCS) (Gibco, Thermo Fisher Scientific, USA) and 2 mM L-glutamine Sigma (Sigma-Aldrich, Germany) (complete medium), at 37°C, 5% CO_2_ and 100% humidity. Raji cell line was kept in culture for less than 2 consecutive months and routinely tested for positivity to mycoplasma.

### 2.4 PBMC *in vitro* stimulation

One hundred thousand PBMC/well were seeded in round-bottomed 96-well plates in complete medium with 1% Penicillin/Streptomycin and co-cultured with γ-irradiated (3.5 Gy) Raji cells, opsonized or not with 1 µg/1x10^6^ rituximab (kindly provided by Dr. Christian Klein, Roche Innovation Centre Zurich, Schlieren, Switzerland) (Effector: Target Ratio = 3:1), for different time lengths. In some experiments, cells were pre-treated with 100 nM rapamycin (cat. R0395-1MG, Sigma) for 2 hrs, and the inhibitor was maintained throughout the culture.

For plate-bound antibody stimulation, 24-well plates were incubated with 10 µg/ml goat anti-mouse IgG F(ab’)_2_ fragment (GaM) (cat. 55487, Cappel, Ohio, USA) for 18 hrs at 4°C in PBS; afterwards, plates were washed twice in PBS and coated with 5 µg/ml αCD16 mAb (clone: B73.1) for 3 hrs at 37°C. PBMC were added (1-1.5x10^6^ cell/well) either in the presence or absence of human recombinant IL-15 (5 ng/ml, Peprotech, UK) for different time lengths.

To analyze IFN-γ production, PBMC were cultured with γ-irradiated Raji cells, opsonized or not with rituximab (3:1 Effector: Target ratio), for 5 hrs at 37°C in the presence of 50 µM Monensin (Golgi-stop; cat.#: M5273, Merck), as previously described (27). Brefeldin A (10 µg/mL, cat.#: B7651, Merck) was added after the first hour of stimulation. In some experiments, PBMC were pre-incubated with 40 mM L-amino acid transport blocker 2-aminobicyclo[2.2.1]heptane-2-carboxylic acid (BCH) (cat.#: A7902-250MG, Sigma), or with 100 nM rapamycin (cat.#: R0395-1MG, Sigma) for 2 hrs. Metabolic inhibitors were present throughout stimulation. Percent of inhibition was calculated as: (1 – the ratio between the percentage of IFN-γ-producing cells in the presence of the inhibitor over the percentage of IFN-γ-producing cells in the presence of the vehicle) x 100. For BCH inhibition, stimulation was carried out in RPMI 1640 medium supplemented with 10% dyalized FCS (cat.#: A3382001, Thermofisher) and 1% L-glutamine.

### 2.5 Assessment of S6 phosphorylation status

PBMC were pretreated overnight at 37°C with 10 ng/ml of human recombinant IL-15 (Peprotech, #200-15) in complete medium. Cells were then washed and incubated with APC-Vio770-conjugated anti-CD56 (Clone: REA 196, cat.#: 130-114-548) and PerCP-Vio700-conjugated anti-CD3 (Clone: BW264/56, cat.#:130-113-132) antibodies (all from Miltenyi Biotech) for 25 minutes on ice. After washing, samples were incubated with the minimum saturating dose (1µg/1x10^6^ cells) of anti-CD16 (clone B7.31) mAb for 20 minutes on ice, washed and resuspended in pre-warmed RPMI1640 medium. Goat F(ab’)_2_ fragment anti-mouse IgG (H+L) (GaM) (Jackson ImmunoResearch Laboratories, #115-006-003) at 0.6 µg/1x10^6^ cells, was added for different time lengths at 37°C. Stimulation was stopped by the addition of Intracellular Fixation & Permeabilization Buffer Set commercial kit (# 00-5523-00, eBioscience, Thermo Fisher Scientific), according to manufacturer’s instructions, centrifuged for 5 minutes at 1400rpm at 4°C and stained with PE-conjugated anti-pS6 ribosomal protein (S235/236) rabbit monoclonal antibody (clone: D57.2.2E, Cell Signaling Technology, #5316S) and FITC-conjugated anti-FcεRIγ subunit polyclonal antibody (Merck, # FCABS400F). Cell samples were acquired on a FACSCanto II (BD Bioscience) and analyzed with FlowJo v.9.3.2 (TreeStar) software.

### 2.6 Kynurenine uptake evaluation

Kynurenine (K8625, Sigma, Germany) was resuspended at 800 µM in Hank’s Balanced Salt Solution (HBSS, cat.#: H8264-500ML, Sigma, Germany) and pre-warmed at 37°C. After surface staining, PBMC were washed with HBSS and incubated with 200 µM Kynurenine for 5 min at 37°C in HBSS. Uptake was stopped by adding PFA (to a final concentration of 1%) for 30 min at RT. After fixation, samples were permeabilized with a 0.05% Triton X-100-containing PBS solution for 20 min at RT, and subjected to intracellular staining. In some experiments, samples were pre-incubated with 40 mM BCH in HBSS, for 2 hrs and then stained for surface markers. Afterwards, samples were washed and incubated with Kynurenine, in the presence of 40 mM BCH for 5 minutes. Kynurenine and BCH were replaced with an equal volume of HBSS in control samples. Kynurenine signal was detected with 405 nm laser, 450/50 BP filter and fluorescence minus one (FMO, w/o Kynurenine) sample was used as negative control. Percent of inhibition of Kynurenine uptake was calculated as: (1 – the ratio between net mFI of intracellular kynurenine in the presence of BCH over net mFI of intracellular kynurenine in the presence of the vehicle) x 100. Cell sample fluorescence signals were acquired with FACSCanto II (BD Biosciences, Franklin Lakes, NJ, USA) and analyzed with FlowJo v10.9.0 (Becton-Dickinson Biosciences, San Jose, CA, USA) software (modified from ref. 63).

### 2.7 Immunostaining and flow cytometric analysis

Freshly-isolated or cultured PBMC underwent surface staining for 25 minutes at 4°C with saturating concentrations of the following fluorophore-conjugated antibodies: anti-CD3 PerCP-Vio700 (clone: BW264/56, cat.#:130-113-132), anti-CD56 APC-Vio770 (clone: REA 196, cat.#: 130-114-548), anti-CD16 PE-Vio770 (clone: REA 423, cat.#: 130-113-394), anti-NKG2C-PE (clone: REA 205, cat.#: 130-119-776), anti-CD57 APC (clone: REA 769, cat.#: 130-111-811) or anti-CD57 PE-Vio770 (clone REA 769, cat.#: 130-111-812), anti-CD98 APC (clone REA 387, cat.#: 130-127-296), isotype control mouse IgG1 APC (clone: REA 205, cat.#: 130-113-196), all from Miltenyi Biotec (Germany); anti-CD71 Alexa Fluor 647 (clone: OKT9, cat.#: 566724) and anti-GLUT1 Alexa Fluor 647 (clone: 202915, cat.#: 566580) were from BD Pharmingen (New Jersey, USA). After surface staining, cells were fixed with 2% PFA (Merck, Germany) for 15 min at RT, permeabilized with PBS with 0.05% Triton X-100 for 20 min at RT and subjected to intracellular staining for 30 min at 4°C with saturating concentrations of the following antibodies: FcεRIγ FITC (rabbit, polyclonal, cat.#: FCABS400F, Merck Millipore) and IFN-γ APC (clone: B27, cat.#: 554702, BD Pharmingen, New Jersey, USA). Cells were washed with a PBS solution containing 2% FCS and 2 mM EDTA disodium salt (washing buffer) after each step.

Live lymphocytes were selected according to FSC-A and SSC-A physical parameters, doublets were excluded based on FSC-H and FSC-A, and memory and conventional NK cell subsets were identified as depicted in **Supplementary Figure S1**. Positivity marker was set according to isotypic control sample (<0.7% positive cells).

### 2.8 Statistical analysis

Statistical analysis was performed with Prism v9.5.0 (GraphPad, San Diego, CA, USA) software package. Wilcoxon signed rank, Mann-Whitney, and ANOVA for repeated measures tests were used, as appropriate. All *p* values were subjected to a significance threshold of 0.05.

## 3 Results

### 3.1 FcεRIγ^-^ memory NK cells display a distinct expression pattern of nutrient transporters *ex vivo*

Although some metabolic peculiarities have been described in memory NK cells (12, 19), the asset of nutrient transporters has not been defined yet. We have initially analyzed the expression of CD98 neutral amino acid antiporter and CD71 transferrin receptor on freshly isolated peripheral blood (PB) NK cell populations of healthy individuals with an expanded FcεRIγ^-^ NK cell pool (see Materials and Methods).

**Figure 1:**
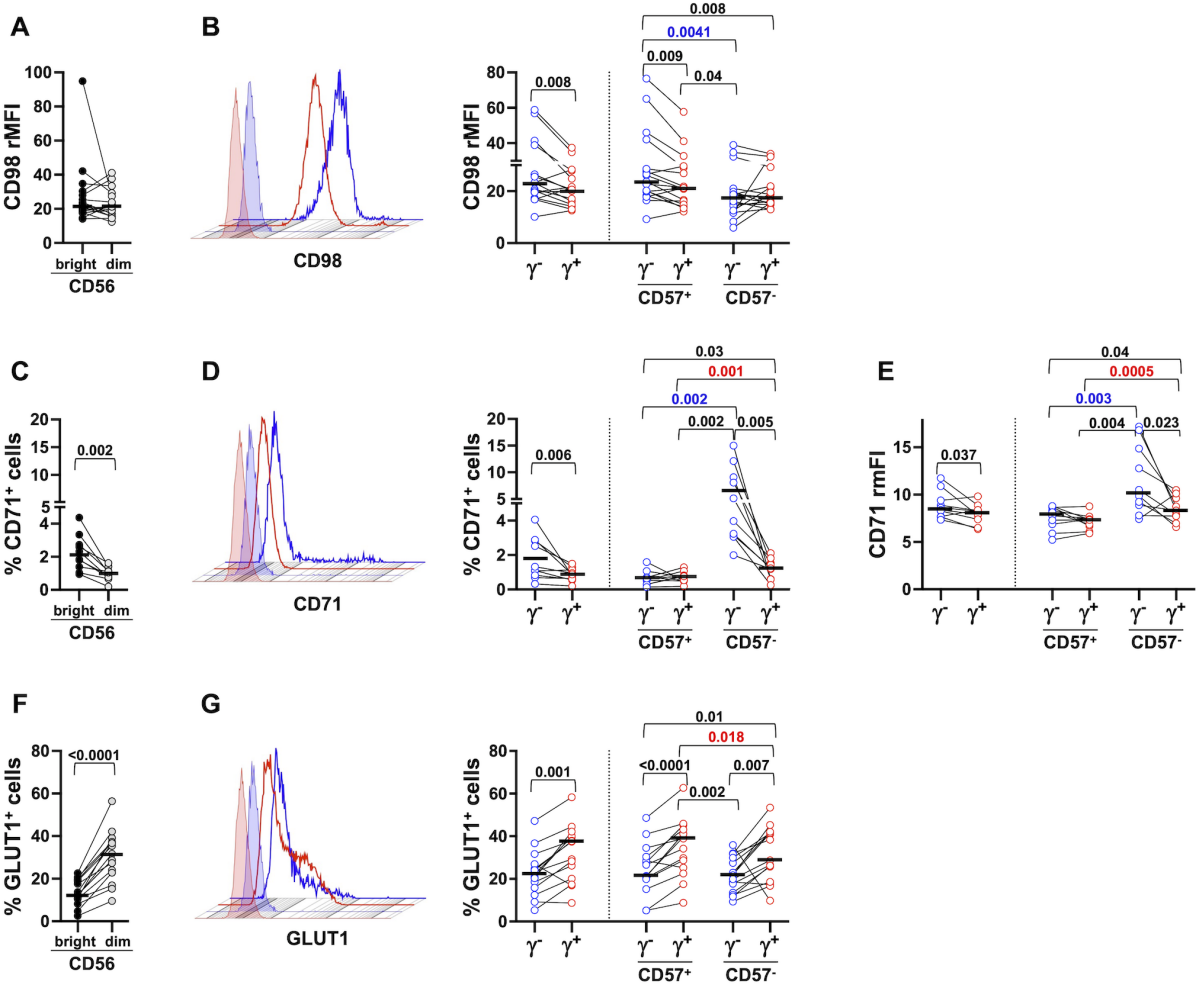
Distinct nutrient transporter expression pattern in peripheral blood memory and conventional NK cell subsets. The expression of CD98 (**A, B**, 17 healthy donors), CD71 (**C–E**, 10 healthy donors) and GLUT1 (**F, G**, 15 healthy donors) was evaluated on freshly isolated CD56^bright^ (dark grey) and CD56^dim^ (light grey) **(A, C, F)** NK cells, on memory (γ^-^, blue circles) and conventional (γ^+^, red circles) CD56^dim^ populations and their respective CD57^+^ and CD57^-^ subsets (**B, D, G**, right panels). Representative histograms of CD98, CD71 and GLUT1 expression in memory (blue) and conventional (red) NK cells are depicted (**B, D, G**, left panels, respectively). Positivity marker was set according to isotypic control sample. CD98 relative Mean Fluorescence Intensity (rMFI) was calculated as the ratio between CD98 MFI and control IgG MFI **(A, B)** CD71 relative median Fluorescence Intensity (rmFI) was calculated as the ratio between CD71 mFI and IgG control mFI **(E)**. Bars represent median values. *p* values of pairwise comparisons between CD56^bright^ and CD56^dim^ (black), memory and conventional subsets (black), and within memory and conventional populations (red and blue, respectively), were calculated with Wilcoxon and ANOVA for repeated measures tests as appropriate.

CD98 was expressed on all PB NK cells, without any significant differences between CD56^dim^ and CD56^bright^ NK cells (**Figure 1A**), in accordance with a previous report (59). CD56^dim^ memory NK cells displayed significantly higher levels of CD98, that were specifically attributable to the more differentiated (CD57^+^) fraction; indeed, the expression of the antiporter did not appreciably differ between memory and conventional (FcεRIγ^+^) CD57^-^ NK cells (**Figure 1B**, right panel).

CD71 transferrin receptor was poorly expressed on mostly resting PB NK cells, with CD56^bright^ displaying higher levels than CD56^dim^ (**Figure 1C**), as previously reported (59). CD71 was significantly more expressed on memory NK cells, limited to the CD57^-^ fraction, when evaluated as either percentage of positive cells and intensity (**Figure 1D**, right panel, and **Figure 1E**, respectively). Of note, CD57^-^ conventional cells displayed higher expression of CD71, as compared to their CD57^+^ fraction (**Figure 1D**, right panel, and **Figure 1E**), in accord with the previously reported higher proliferative competence of less mature population (60).

In marked contrast, the major glucose transporter GLUT1, present on a higher fraction of CD56^dim^ than CD56^bright^ PB NK cells (**Figure 1F**), was more abundantly expressed on conventional NK cells, independently of their functional maturation stage (**Figure 1G**). Taken together, these data indicate that circulating FcεRIγ^-^ memory NK cells display a distinct pattern of nutrient transporters, characterized by higher expression of CD98 and CD71 and decreased levels of GLUT1, with respect to conventional ones. The data also suggest the impact of functional maturation state on the *in vivo* distinctive profile of memory population; indeed, enhanced expression of neutral amino acid antiporter positively associates with a more advanced stage of differentiation, while more elevated expression of transferrin receptor characterizes less mature cells.

### 3.2 CD16-dependent interaction with antibody-opsonized target cells upregulates CD98 and CD71 nutrient transporters on memory and conventional NK cells

CD16 engagement by contact with IgG-coated targets triggers the full effector program of human CD56^dim^ NK cells (22–26), in the antibody-dependent killer activation (ADKA) modality. Moreover, hyperresponsiveness to CD16-initiated signals is the most prominent functional feature of HCMV-dependent FcεRIγ^-^ memory cells (1–3, 5–8, 14–20). However, the capability of CD16-dependent activation to induce a persistent metabolic reprogramming is still mostly unexplored.

**Figure 2:**
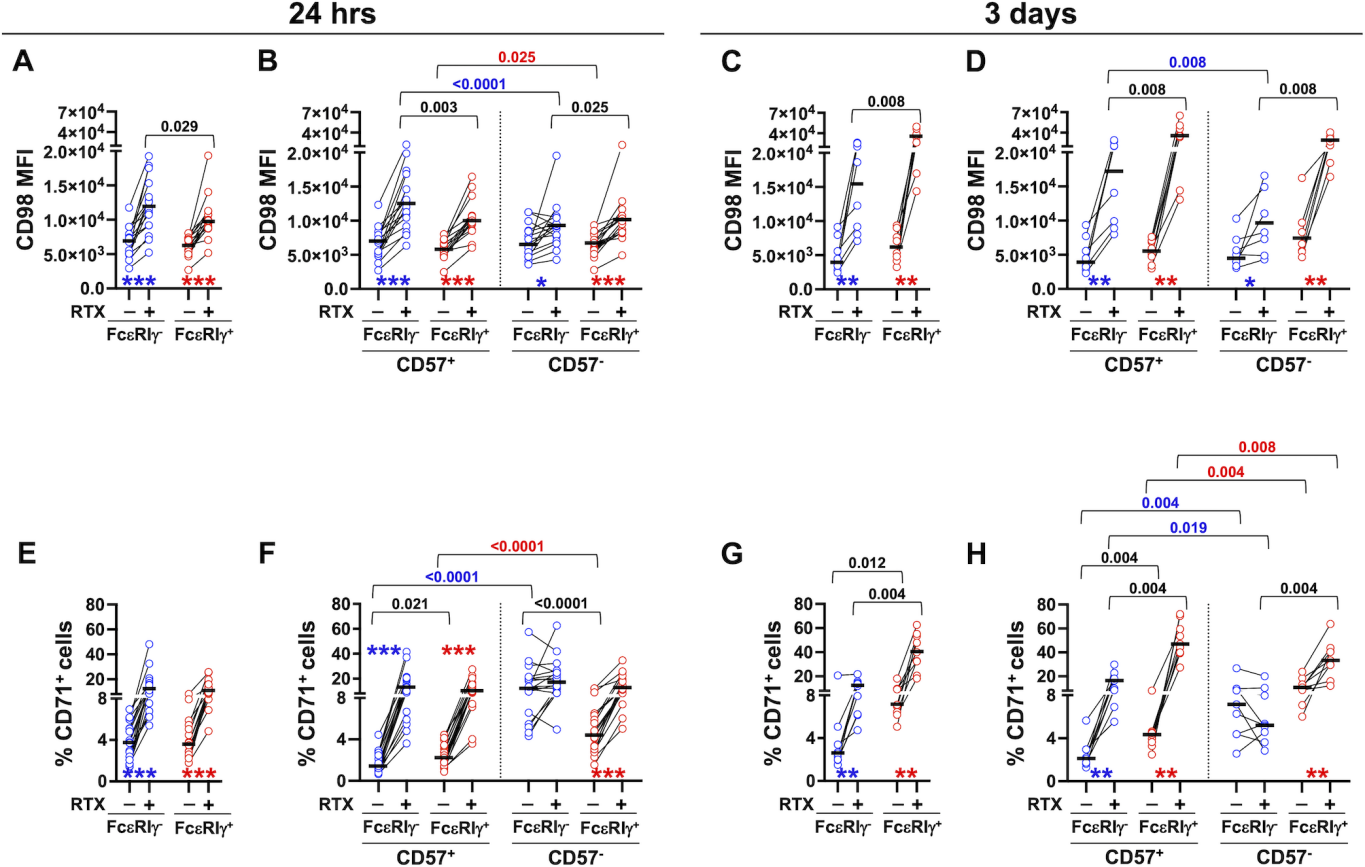
Stimulation with mAb-opsonized target cells induces the upregulation of CD98 and CD71 nutrient transporters in memory and conventional NK cell subsets. PBMC were stimulated with B lymphoma Raji target cells in the presence (+) or absence (-) of rituximab (RTX) for 24 hrs (**A, B**, 16 healthy donors and **E, F**, 18 healthy donors) or 3 days (**C, D**, 8 healthy donors and **G, H**, 9 healthy donors); CD98 **(A–D)** and CD71 **(E–H)** expression was evaluated in memory (FcεRIγ^-^, blue circles) and conventional (FcεRIγ^+^, red circles) NK cell populations **(A, C, E, G)**, and in their respective CD57^+^ and CD57^-^ subsets **(B, D, F, H)**. Stimulations at different time points were carried out on distinct groups of individuals. Bars represent median values. *p* values of pairwise comparisons between memory and conventional subsets (black), and within memory and conventional populations (red and blue, respectively), were calculated with Wilcoxon non-parametric test. Symbols in graphs refer to the comparison between presence *vs* absence of RTX: *p<0.02, **p<0.008, ***p<0.0001.

Twenty-four hour co-culture with rituximab (RTX)-opsonized Raji B cell lymphoma targets induced robust upregulation of CD98 and CD71 nutrient transporters on memory and conventional NK pools (**Figures 2A, E**). The response was largely limited to CD57^+^ memory cells, while it occurred irrespectively of the maturation status in the conventional NK cell pool (**Figures 2B, F**). Interestingly, CD98 levels were higher on ADKA-stimulated CD57^+^ memory NK cells, as compared with the conventional counterpart (**Figures 2A, B**), thus reproducing the difference observed in *ex vivo* populations (**Figure 1B**). At variance, the percentage of CD71^+^ cells was comparably increased in memory and conventional NK cell pools by 24-hr stimulation with RTX-opsonized targets (**Figure 2E**). In the absence of opsonizing mAb, CD57^-^ memory NK cells exhibited the highest expression of CD71, that was unaffected by CD16 engagement (**Figure 2F**); in the same conditions, both CD57^-^ fractions of memory and conventional NK cells showed higher expression of CD71, as compared to their more terminally differentiated counterparts, similarly to what observed *ex vivo* (**Figure 2F**, compared with **Figure 1D**).

More prolonged (3 days) stimulation with mAb-opsonized targets sustained the upregulation of both nutrient transporters in FcεRIγ^-^ and FcεRIγ^+^ populations, as compared with co-culture in the absence of antibody (**Figures 2C, G**). At this time point, CD98 and CD71 reached significantly higher levels in conventional NK cells, as compared to memory counterpart, in contrast with what observed in a short-term stimulation (compare **Figure 2A** with **Figure 2C**, and **Figure 2E** with **Figure 2G**). Similarly to what observed after 24-hr stimulation, CD16-dependent upregulation of nutrient transporters was largely confined to CD57^+^ memory fraction, while robustly occurring in all conventional cells (**Figures 2D, H**). Of note, CD57^-^ memory and conventional NK cells had higher levels of CD71 expression in control cultures, as compared to their CD57^+^ counterparts, thus maintaining the profiles observed in either freshly isolated and short-term stimulated populations. Overall, these data firstly show that human CD56^dim^ NK cells rapidly (24 hrs) and persistently (up to 3 days) upregulate CD98 and CD71 nutrient transporters in response to contact with mAb-opsonized targets. Additionally, the efficiency of ADKA stimulation to promote the regulation of nutrient transporters strictly depends on functional maturation stage, as marked by CD57 expression, in FcεRIγ^-^ memory, but not FcεRIγ^+^ conventional population; indeed, the upregulation of transporters on memory NK cells was stronger on more differentiated fraction, while more limited (CD98) or absent (CD71) in the CD57^-^ subset, at both time points. Kinetic analysis indicates the memory and conventional pools differently respond to the persistence of Ab-dependent stimulation, as short-term stimulation efficiently supported the higher, *ex vivo*, expression of CD98 on memory cells, but this advantage was lost upon chronic stimulation. On the other hand, the higher expression of CD71 observed on freshly isolated CD57^-^ memory NK cells persisted in the absence of opsonizing antibody, at shorter culture time point, while it was not supported by interaction with Ab-coated targets. Collectively, the data suggest that *in vivo* contact with IgG-coated cells may contribute to the enhanced expression of CD98, while other signals are responsible for sustaining the higher expression of transferrin receptor in freshly isolated memory NK cells.

Further stratification of CD56^dim^ NK cells revealed that the NKG2C/CD57 coexpressing subset tendentially displayed the highest level of both nutrient transporters, within either memory and conventional pools, at either time point of stimulation (**Supplementary Figures S2A–D**). This evidence supports the hypothesis that it represents a fully mature subset endowed with amplified sensitivity to CD16 engagement, as our group has previously shown for other functional responses (21).

Collectively taken, these data firstly show that effective interaction with antibody-coated targets promotes the upregulation of CD98 and CD71 transporters on human PB CD56^dim^ NK cells. The dynamics of the nutrient transporters in response to ADKA stimulation suggests that *in vivo* pattern of expression, functional maturation stage and persistence of the stimulus are all factors that contribute to the different behaviour of the two nutrient receptors in memory and conventional populations.

### 3.3 Role of direct CD16 crosslinking in the upregulation of CD98 and CD71 expression on memory and conventional NK cells

**Figure 3:**
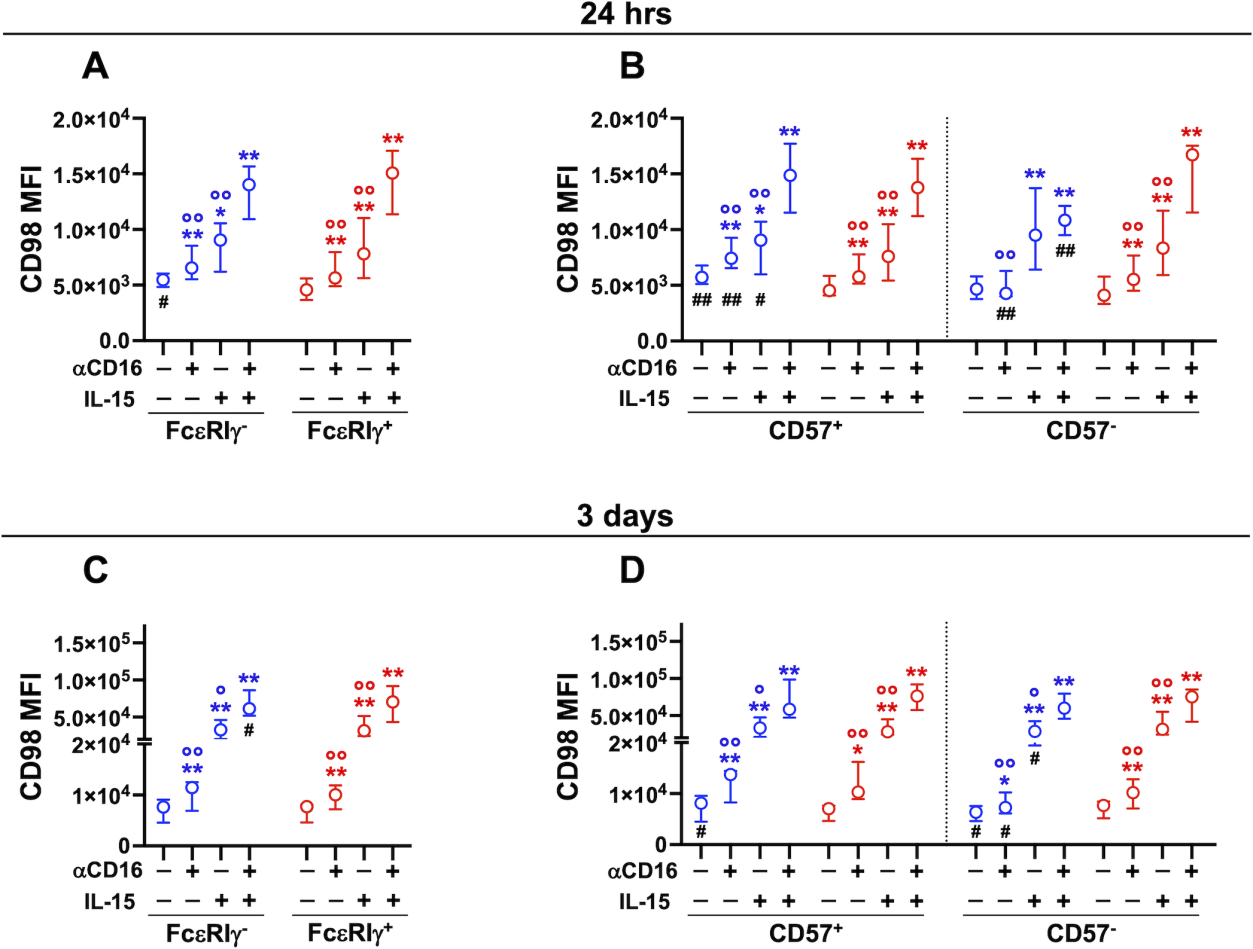
Stimulation with immobilized αCD16 mAb upregulates CD98 on memory and conventional NK cells. Cooperation with IL-15. PBMC were stimulated with plastic-bound αCD16 mAb and/or 5 ng/ml IL-15 for 24 hrs (**A, B**, 8 healthy donors), or 3 days (**C, D**, 7 healthy donors) and CD98 MFI was evaluated in memory (FcεRIγ^-^, blue) and conventional (FcεRIγ^+^, red) NK cell populations **(A, C)**, and in their respective CD57^+^ and CD57^-^ subsets **(B, D)**. Stimulations at different time points were carried out on distinct groups of individuals. Circles and bars represent medians and interquartile ranges, respectively. *p* values were calculated with Wilcoxon and ANOVA for repeated measures tests as appropriate. Symbols in graphs refer to differences of: unstimulated *vs* αCD16, IL-15, or αCD16+IL-15 (*); αCD16 or IL-15 *vs* αCD16+IL-15 combination (°); memory *vs* conventional populations (#). *,°,^#^ *p*<0.05; **,°°,^##^ *p*<0.008.

Several soluble and cell-associated signals may cooperate with CD16-initiated transduction pathways during NK cell interaction with antibody-opsonized targets. We thus assayed the regulation of CD98 and CD71 expression upon direct CD16 crosslinking. Stimulation with plastic-immobilized αCD16 mAb rapidly (24 hrs) and persistently (3 days) promoted the upregulation of CD98 on memory and conventional NK cells (**Figures 3A, C**). CD16-triggered upregulation of CD98 on memory cells at 24 hrs was restricted to the CD57^+^ fraction, and resulted in significantly higher expression of the transporter compared to conventional counterpart (**Figure 3B**). Both distinctive features were abrogated upon more sustained crosslinking of CD16 (**Figure 3D**), similarly to what observed in the ADKA setting (**Figures 2G, H**). Notably, unstimulated CD57^+^ memory cells displayed higher levels of CD98 than their conventional counterpart, in accordance with the profile of freshly isolated cells (**Figures 3B, D**). The pattern and kinetics of CD98 modulation by αCD16 mAb-mediated crosslinking reproduced what observed upon stimulation with mAb-opsonized targets, although to a more limited extent, suggesting that other factors in the co-culture with RTX-opsonized Raji cells may amplify CD16-dependent effect. Low dose IL-15 (5 ng/ml) enhanced CD98 expression on both memory and conventional NK cells (**Figures 3A, C**), irrespectively of the functional maturation stage (**Figures 3B, D**). Notably, CD16 crosslinking in the presence of IL-15 led to a further enhancement of CD98 expression in both memory and conventional populations (**Figures 3A, C**). At shorter stimulation timepoint, this cooperation occurred on conventional NK cell subsets independently of CD57 expression, while it was limited to CD57^+^ memory NK cells, in accordance with the lower sensitivity of the CD57^-^ subset to CD16 crosslinking (**Figures 3B, D**). Collectively, these data indicate that CD16 crosslinking is sufficient to promote CD98 upregulation during the interaction with IgG-coated target cells, and suggest that cytokine signals in the microenvironment amplify CD16-dependent regulation of the amino acid antiporter.

**Figure 4:**
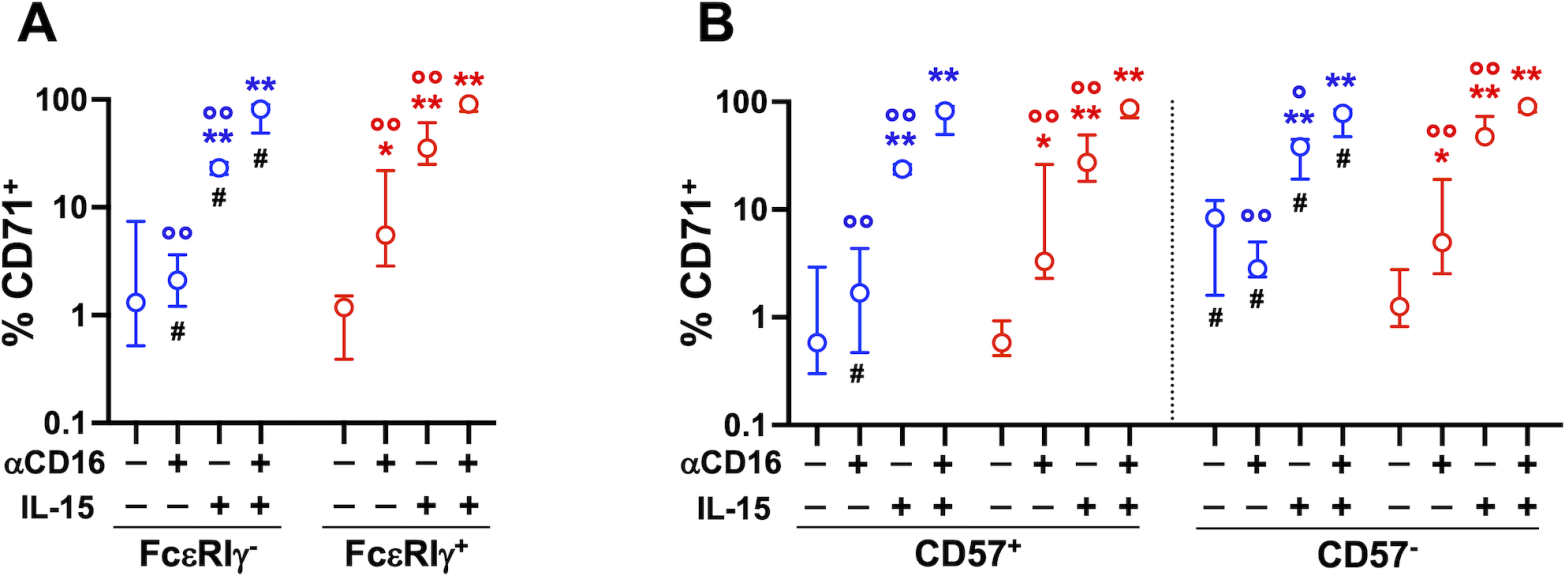
Stimulation with immobilized αCD16 mAb upregulates CD71 on memory and conventional NK cells. Cooperation with IL-15. PBMC were stimulated with plastic-bound αCD16 mAb and/or 5 ng/ml IL-15 for 3 days (7 healthy donors), and the percentage of CD71^+^ cells was evaluated in memory (FcεRIγ^-^, blue) and conventional (FcεRIγ^+^, red) NK cell populations **(A)**, and in their respective CD57^+^ and CD57^-^ subsets **(B)**. Circles and bars represent medians and interquartile ranges, respectively. *p* values were calculated with Wilcoxon and ANOVA for repeated measures tests as appropriate. Symbols in graphs refer to differences of: unstimulated *vs* αCD16, IL-15, or αCD16+IL-15 (*); αCD16 or IL-15 *vs* αCD16+IL-15 combination (°); memory *vs* conventional populations (#). *,°,^#^ *p*<0.05; **,°° p<0.008.

Short-term (24 hrs) CD16 stimulation, in combination or not with low-dose (5 ng/ml) IL-15, did not affect CD71 expression on PB memory and conventional NK cells (De Federicis et al, unpublished observations). However, three-day stimulation with plastic-bound αCD16 mAb induced significant upregulation of transferrin receptor on both CD57^+^ and CD57^-^ conventional NK cells, but not on the respective memory FcεRIγ^-^ counterparts (**Figures 4A, B**). Low-dose IL-15 augmented the percentage of CD71^+^ memory and conventional cells, and potentiated the effect of CD16 receptor crosslinking on all CD56^dim^ subsets, to a comparable extent. Of note, CD71 expression on unstimulated CD57^-^ memory NK cells was significantly higher than on their conventional counterpart, similarly to what observed in *ex vivo* populations (**Figure 4B**). Collectively, these data indicate that CD16 crosslinking is sufficient to induce upregulation of transferrin receptor on conventional NK cells, while memory cells require the presence of additional signals provided during the interaction with IgG-opsonized target cells.

### 3.4 CD16-triggered mTORC1 activation regulates CD98 and CD71 expression on CD56^dim^ NK cells

**Figure 5:**
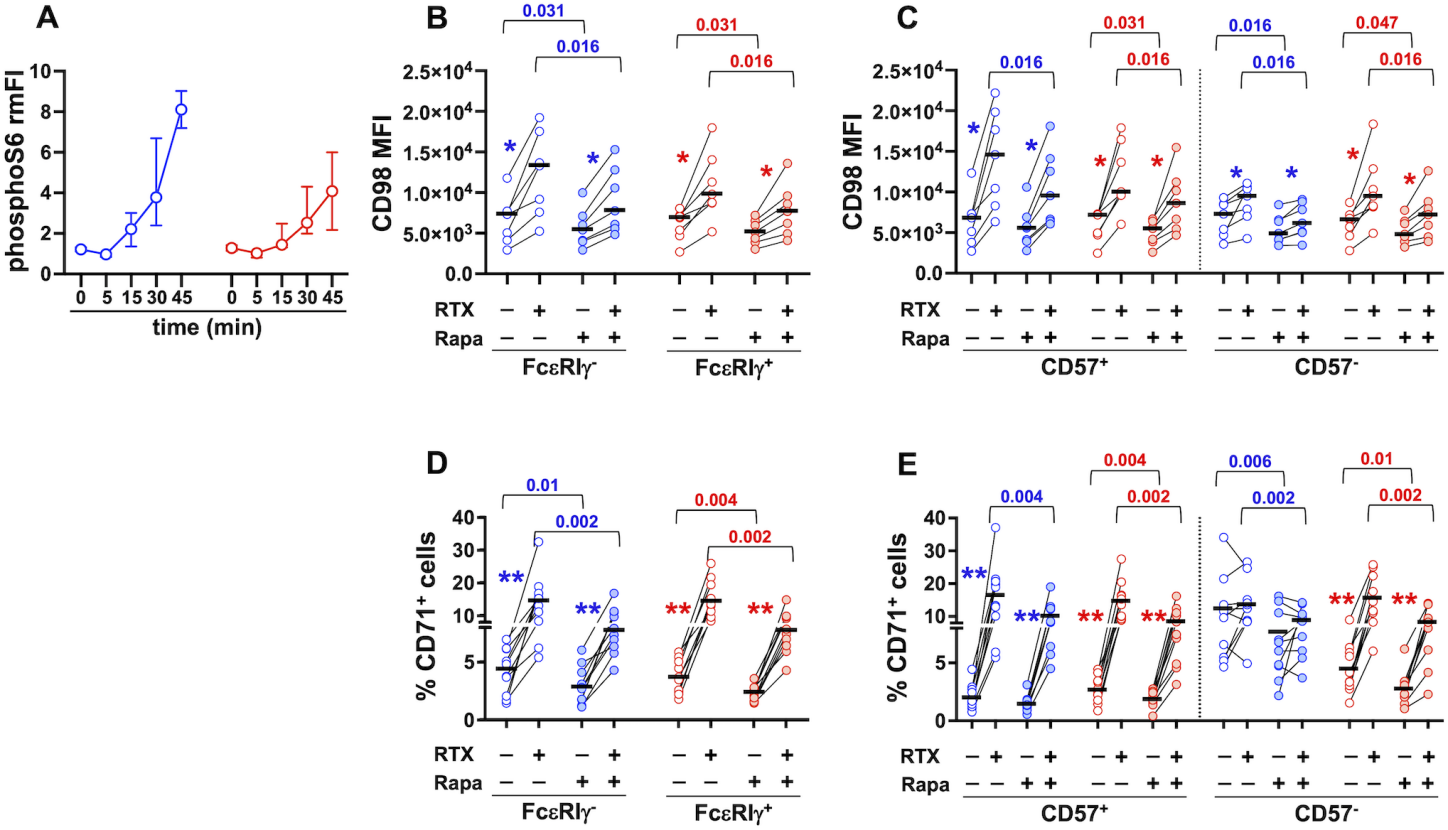
CD16-triggered upregulation of CD98 and CD71 depends on mTORC1 activation. **(A)** PhosphoS6 kinetics was evaluated in memory (blue) and conventional (red) CD56^dim^ NK cells upon CD16 engagement. Relative median Fluorescence Intensity (rmFI) was calculated as the ratio between stimulated (anti-CD16+GaM) and unstimulated (GaM only) sample, for each time point. Bars represent median values and interquartile intervals. CD98 Mean Fluorescence Intensity (MFI) (**B, C**, 7 individuals) and percentage of CD71^+^ cells (**D, E**, 9 individuals) were evaluated upon 24 hr stimulation with RTX-opsonized or not-opsonized Raji targets, in the presence (shaded symbols) or absence (empty symbols) of 100 nM Rapamycin. Bars represent median values. Pairwise comparisons were performed with non-parametric Wilcoxon test. *p* values of the comparisons between untreated and Rapamycin-treated samples of memory and conventional populations are reported. Symbols in graphs refer to the comparisons between presence *vs* absence of RTX: *p=0.016, **p=0.002.

Cytokine-induced upregulation of CD98 and CD71 in human NK cells was shown to involve mTORC1 activation (61). Interestingly, CD16 engagement acutely promoted stronger activation of mTORC1 in FcεRIγ^-^ memory NK cells with respect to FcεRIγ^+^ conventional population (19). Here we show that CD16-dependent activation of mTORC1, evaluated by phosphorylated S6 levels, was maintained at higher levels in memory NK cells in an extended kinetics (**Figure 5A**). Interestingly, Rapamycin, at a dose that specifically inhibits mTORC1 activation, reduced the extent of ADKA-induced upregulation of CD98 (**Figures 5B, C**) and CD71 (**Figures 5D, E**) on memory and conventional NK cell subsets. Of note, the presence of Rapamycin consistently reduced the levels of both transporters also in cultures with not-opsonized Raji cells, with the only exception of CD57^+^ memory NK cell subset (**Figures 5C, E**), suggesting that mTORC1 activation is also involved in regulating basal nutrient transporter levels.

These data demonstrate that the activation of the mTORC1 pathway is critically involved in the capability of CD16 engagement to upregulate two important nutrient transporters on human NK cells.

### 3.5 CD98-mediated amino acid transport regulates CD16-triggered effector functions of memory and conventional NK cells. Involvement of mTORC1 pathway

**Figure 6:**
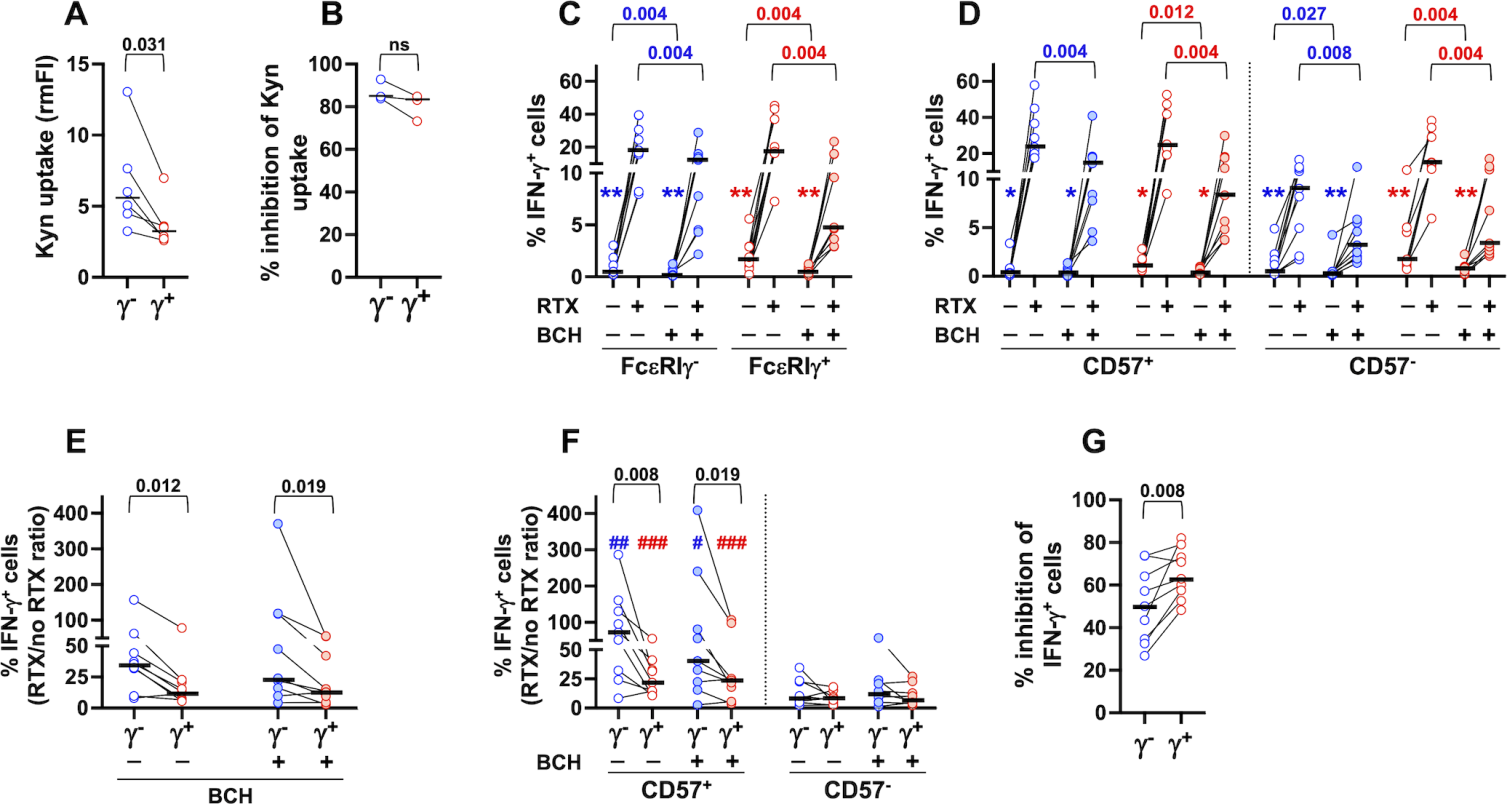
CD98/SLC7A5-dependent neutral amino acid intake is required for optimal CD16-dependent production of IFN-γ. **(A)** Kynurenine uptake was analyzed in peripheral blood CD56^dim^ memory (γ^-^, blue symbols) and conventional (γ^+^, red symbols) NK cells of 6 healthy donors by flow cytometry. Relative median fluorescence intensity (rmFI) was calculated with respect to FMO (w/o Kynurenine) sample. **(B)** Percentage of inhibition of Kynurenine uptake by treatment with BCH (3 healthy donors), evaluated as described in the relevant Materials and Methods section. **(C, D)** Percentage of IFN-γ-producing cells in response to stimulation with rituximab (RTX)-opsonized, or not, Raji lymphoma targets was assayed in the presence (shaded symbols) or absence (empty symbols) of BCH inhibitor (9 individuals) in memory (blue) and conventional (red) populations **(C)** and their CD57^+^ and CD57^-^ subsets **(D)**. Symbols in graphs refer to the comparisons between presence *vs* absence of RTX: *p=0.008, **p=0.004 **(C, D)**. The percentage of IFN-γ^+^ cells was always significantly higher in CD57^+^ than CD57^-^ fraction, in both memory and conventional populations, either in the presence or absence of BCH (*p*=0.004, not reported) **(D)**. **(E, F)** CD16-dependent fold increase of IFN-γ producing cells, expressed as the ratio between the presence *vs* absence of rituximab (RTX/no RTX), was evaluated in the presence (shaded symbols) or absence (empty symbols) of BCH in memory and conventional NK cells **(E)** and their respective CD57^+^ and CD57^-^ subsets **(F)**. Symbols in graph refer to the comparisons between CD57^+^ *vs* CD57^-^: ^#^p<0.05, ^##^p=0.008, ^###^p=0.004 **(F)**. **(G)** Percent of BCH-dependent inhibition of IFN-γ producing cells was evaluated in CD16-stimulated memory (FcεRIγ^-^, blue) and conventional (FcεRIγ^+^, red) CD56^dim^ NK cells, as described in the relevant Materials and Methods section. Bars represent median values. Non-parametric Wilcoxon test was used for all pairwise comparisons. Black *p* values refer to the comparisons between memory and conventional subsets; blue and red *p* values refer to the comparisons within memory and conventional populations, respectively.

To evaluate whether higher expression of CD98 on PB memory NK cells correlates with enhanced neutral amino acid intake by its associated SLC7A5 light chain, we took advantage of Kynurenine, a violet-emitting tryptophan metabolite that is imported by SLC7A5/CD98 heterodimeric antiporter (58). In line with the higher expression of the antiporter, memory NK cells displayed a larger uptake of Kynurenine (**Figure 6A**); the presence of BCH, a selective inhibitor of the antiporter, efficiently abrogated Kynurenine uptake in both memory and conventional populations (**Figure 6B**).

We evaluated the impact of CD98/SLC7A5-dependent amino acid transport on CD16-triggered IFN-γ production upon stimulation with RTX-opsonized Raji cells. The presence of BCH led to a significant decrease of the percentage of IFN-γ-producing memory and conventional NK populations (**Figure 6C**), with a comparable effect on their CD57^+^ and CD57^-^ respective subsets (**Figure 6D**). BCH treatment also largely affected the low production of IFN-γ in the absence of opsonizing antibodies (**Figures 6C, D**). As a consequence, the amplified response of FcεRIγ^-^ memory cells (7, 8, 14, 15, 18, 19, 21), as well as the enhanced response of CD57^+^ cells to CD16 stimulation (28), persist in the presence of the inhibitor, when evaluated as the fold increase in the percentage of IFN-γ-producing cells stimulated by RTX-coated targets (**Figures 6E, F**).

Interestingly, memory NK cells exhibited lower sensitivity to BCH-mediated inhibition of IFN-γ expression (**Figure 6G**), in line with the higher neutral amino acid uptake (**Figure 6A**), suggesting that larger amino acid storage capability contributes to their enhanced responsiveness to CD16 signals.

Collectively, these data firstly show that CD98 amino acid transporter function is required for CD16-dependent IFN-γ production by CD56^dim^ memory and conventional NK cells.

**Figure 7:**
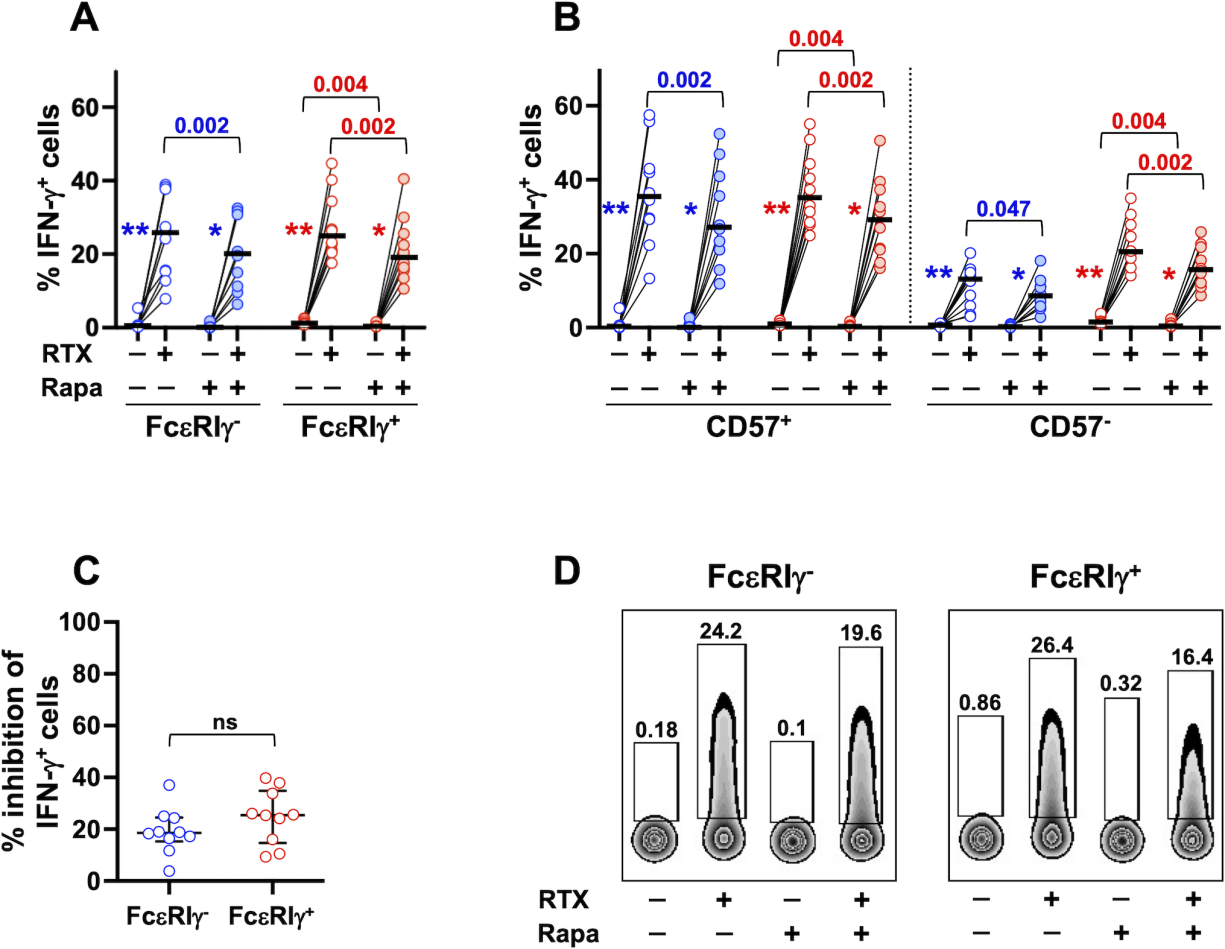
mTORC1 signalling pathway is involved in CD16-dependent IFN-γ production of memory and conventional NK cells. PBMC from 10 healthy donors were stimulated with RTX-opsonized, or not, Raji target cells, in the presence (shaded symbols) or in the absence (empty symbols) of Rapamycin mTORC1 inhibitor. The percentage of IFN-γ-producing cells was evaluated in CD56^dim^ memory (blue) and conventional (red) NK cells **(A)**, and their respective CD57^+^ and CD57^-^ subsets **(B)**. Percent of Rapamycin-dependent inhibition of IFN-γ producing cells was evaluated in CD16-stimulated memory (FcεRIγ^-^, blue) and conventional (FcεRIγ^+^, red) CD56^dim^ NK cells, as described in the relevant Materials and Methods section **(C)**. Bars represent median values. Non-parametric Wilcoxon test was used for all pairwise comparisons. Blue and red *p* values refer to the comparisons within memory and conventional populations, respectively. Symbols in graphs refer to the comparisons between presence *vs* absence of RTX: *p=0.004, **p=0.002. **(D)** Plots depicting IFN-γ-producing memory and conventional NK cells of a representative donor, stimulated with RTX-opsonized, or not, Raji target cells, in the presence or absence of Rapamycin. ns, not significant.

CD98/SLC7A5-dependent import of neutral amino acids, such as leucine, sustains basal activation of mTORC1 and allows its further stimulation by growth factors or cytokines (39, 41–43, 62). Our previous work demonstrated the role of PI3K/mTORC1 axis in CD16-dependent priming of NK cell response to cytokine activation (54). However, the involvement of mTORC1 activation in the CD16-dependent production of IFN-γ is still unclear. Rapamycin treatment reduced CD16-triggered IFN-γ response of memory and conventional NK cells, to a similar and limited extent (**Figures 7A, C, D**), and independently of the functional differentiation stage (**Figure 7B**), suggesting that mTORC1 pathway is only partially involved in CD16-triggered IFN-γ secretion. Moreover, the significantly higher capability to produce IFN-γ was still apparent in Rapamycin-treated memory cell samples, when evaluated as the fold increase in the percentage of IFN-γ-producing cells stimulated by RTX-coated targets (data not shown). Notably, inhibition of CD98/SLC7A5 antiporter functionality by BCH more markedly impacted CD16-triggered IFN-γ production (**Figure 6G**) than Rapamycin effect (**Figure 7C**), in both memory (*p*=0.0002) and conventional (*p*<0.0001) NK populations. These data strongly suggest that neutral amino acid intake sustains CD16-triggered IFN-γ expression through both mTOR-dependent and -independent pathways. Of note, CD16-independent (co-cultures with not-opsonized Raji cells) production of IFN-γ by conventional, but not memory, CD57^+^ and CD57^-^ subsets was also impaired by rapamycin treatment (**Figures 7A, B, D**).

Our results demonstrate the contribution of mTORC1 activation in the CD16-triggered metabolic reprogramming and functional response, and suggest that the peculiar nutrient transporter profile contributes to memory NK cell hyperresponsiveness to CD16 engagement.

## 4 Discussion

This work firstly demonstrates that human memory CD56^dim^FcεRIγ^-^ NK cells are endowed with a specific nutrient expression pattern. We also describe for the first time the capability of CD16 engagement to dynamically modulate the expression of CD98/SLC7A5 heterodimeric neutral amino acid antiporter and CD71 transferrin receptor on human NK cells through a mTORC1-dependent pathway. Relevantly, our results indicate that functional maturation stage, persistence of CD16 engagement and cooperation with cytokine signals interplay in finely regulating the expression of CD98 and CD71 on memory and conventional NK cell populations. Finally, we show that CD98 functional activity is required for the optimal CD16-dependent production of IFN-γ, through both mTORC1-dependent and -independent mechanisms, thus supporting the link between metabolic capacity and functional profile of a specific population (56).

NK cell lifespan, proliferative potential, and effector functions are largely influenced by cell metabolism and nutrient transporter profile, which depend on the specific subset, maturation level, activation status, and body district (33, 34, 45, 46, 48, 56, 61, 63). Moreover, evidence for NK cell metabolic flexibility (49) indicates their capability to react to environmental stimuli by changing their metabolism, in order to optimize their response (47). Nevertheless, the mechanisms that regulate metabolic reprogramming of NK cells in response to environmental signals are poorly understood yet.

Although basal glycolytic and oxidative metabolic activity, mitochondrial fitness, and anti-apoptotic Bcl-2 protein content were reported to be distinctively higher in either FcεRIγ^-^ and/or NKG2C^+^CD57^+^ memory NK cells (12, 15, 16, 21, 45, 59), the contribution of intracellular energetic pathways to their enhanced survival and functional response upon interaction with antiviral-, antitumor-, or autoantibody-coated target cells is still unclear (1–8, 14–21, 47). Whether these CD16-dependent distinctive features are supported by adequate nutrient intake is still object of investigation.

We show that memory NK cells express significantly higher levels of CD98 and CD71 nutrient transporters than the conventional counterpart *ex vivo*. The enhanced expression of such transporters tightly depended on memory functional maturation level; indeed, CD98 was hyperexpressed on CD57^+^ cells, while CD71 expression, an indicator of proliferative competence, was selectively higher on CD57^-^, less functionally differentiated, fraction. On the opposite, the major glucose-transporter GLUT1, was more abundantly expressed on conventional NK cells.

Our data firstly show that 24hr stimulation with rituximab-opsonized Raji lymphoma cells (ADKA modality) enhanced CD98 and CD71 transporter expression on CD56^dim^ NK cells, and the upregulation persisted up to three days of sustained contact with mAb-opsonized targets. Our analysis provides evidence that the persistence of CD16 engagement and the functional maturation stage differently affect the dynamics of individual transporters, in memory and conventional populations. CD98 upregulation at 24 hr stimulation was more marked on CD57^+^ memory cells, thus mirroring the pattern of fresh, *ex vivo*, populations. At variance, a more prolonged (3 days) interaction with mAb-opsonized targets induced a stronger upregulation of the antiporter in conventional NK cells, independently from their degree of differentiation. Direct CD16 crosslinking by plastic-bound mAb reproduced a qualitatively similar pattern of CD98 modulation, underscoring a prominent role of CD16-initiated signalling pathways in the upregulation of CD98. In this more simplified setting, the presence of low-dose IL-15 amplified CD16-dependent upregulation of the antiporter in both memory and conventional NK cells. Noteworthy, CD57^-^ memory, but not conventional, NK cells displayed a lower sensitivity to CD16 stimulation in both experimental settings.

ADKA stimulation also resulted in the upregulation of CD71 transferrin receptor, the main responsible for iron uptake, to an extent that was initially comparable in memory and conventional NK cells, and became more pronounced in conventional population at a longer time point. The upregulation of CD71 was limited to more functionally mature memory cells, while it occurred independently of maturation status in conventional ones; of note and in accord with the *ex vivo* pattern, CD71 expression on CD57^-^ memory cells was highest in control cultures. These data suggest that, differently from what observed for CD98 antiporter, CD16 engagement by IgG-coated cells may not be sufficient, by itself, to sustain the enhanced levels of CD71 of *ex vivo* memory cells, and that cooperating signals are required. The amount and/or quality of these signals appear to depend on the functional maturation stage (CD57^+^ vs CD57^-^), a known factor that affects the extent of responsiveness to CD16 stimulation (28). This hypothesis is supported by the evidence that stimulation with plastic-immobilized anti-CD16 mAb, by itself, induced CD71 upregulation on conventional, but not on memory NK cells, although the addition of low-dose IL-15 significantly cooperated with CD16 engagement on both memory and conventional subsets. In this regard, the capability of CD2 co-engagement to amplify CD16-initiated signalling pathways and effector responses of memory FcεRIγ^-^ NK cells was previously reported (19). As CD71 transferrin receptor expression is considered a measure of proliferative competence, these findings may contribute to the comprehension of the mechanisms that regulate expansion and stability of the memory NK cell pool *in vivo*.

Our results indicate that the time length of contact with antibody-opsonized targets differently affects the modulation of nutrient transporters on memory and conventional NK cells. The higher (CD98) or comparable (CD71) expression of memory cells at shorter time of stimulation was lost in favor of conventional ones upon more prolonged ADKA stimulation. The underlying mechanisms are unknown, but chronic NKG2C engagement was previously shown to promote memory NK cell exhaustion (64).

The ability of several cytokines to enhance the expression of CD98 and CD71 on human NK cells has been widely reported (53, 59, 61, 63, 65). Our study represents the first evidence of the capability of CD16, an activating receptor that globally enables all NK cell effector functions, to rewire nutrient transporter expression on CD56^dim^ NK cells. We also dissected the role of cell-dependent (stage of functional maturation, memory or conventional lineage) and stimulus-related (persistence of CD16 engagement, cooperation with cytokines) factors on this response. The expression of NKG2C activating receptor coupled with CD57 marker of functional competence has been employed to identify memory/adaptive lineage in several studies (12, 29); a previous report showed that NKG2C^+^CD57^+^ adaptive NK cells, partially overlapping with FcεRIγ^−^ memory population, displayed higher glucose uptake in response to short-term CD16 triggering (12). Interestingly, our data show that NKG2C/CD57 coexpressing subset generally expressed the highest level of both nutrient transporters upon stimulation with opsonized targets, within either FcεRIγ^−^memory and FcεRIγ^+^ conventional pools. This is in line with a previous study that identified it as a fully mature subset endowed with amplified functional responses to CD16 engagement (21).

We provide evidence that mTORC1 activation controls CD16-triggered hyperexpression of CD98 and CD71 transporters. In this context, CD16 crosslinking promoted sustained S6 phosphorylation, that was tendentially higher in FcεRIγ^-^ memory cells. Of note, rapamycin treatment also reduced CD98 and CD71 expression in co-cultures with not-opsonized target cells, suggesting that mTORC1 also regulates the basal expression of the transporters. The mechanisms responsible for CD16-dependent upregulation of transporters downstream mTORC1 activation are unknown. Interestingly, IRF4 transcription factor was recently shown to orchestrate CD98 and CD71 expression and virus-driven expansion of murine memory NK cells; this transcription factor was more abundantly expressed in NKG2C^+^CD57^+^ cells of HCMV^+^ donors, where it was upregulated by NKG2C plus cytokine stimulation (66). Additionally, CD98 and CD71 genes are known targets of c-Myc (35, 67); in a feed-forward loop, amino acids that are uptaken through CD98/SLC7A5 antiporter sustain mTORC1 activity and further support c-Myc expression (35, 42, 43); indeed, inhibition of SLC7A5 in cytokine-activated NK cells resulted in reduced c-Myc protein levels and mTORC1 signalling (35, 53). Finally, CD98 and CD71 upregulation may also involve post-transcriptional mechanisms (35, 42, 67).

CD16 engagement rapidly upregulates glycolysis and oxidative phosphorylation, which are essential for CD16-triggered IFN-γ production of human NK cells (55). Our results firstly show that CD98/SLC7A5 antiporter activity is required for optimal CD16-triggered IFN-γ production in fresh memory and conventional NK cells. In this context, the higher expression of CD98 on CD57^+^ functionally mature memory NK cells well correlates with enhanced CD16-triggered IFN-γ production, with respect to either conventional counterpart and to CD57^-^ memory fraction. CD98 antiporter function was previously shown to be involved in cytokine-triggered IFN-γ production (61) and essential for NKG2D-dependent IFN-γ production and degranulation in IL-2 primed NK cells (53). Interestingly, memory NK cell IFN-γ production shows a lower sensitivity to BCH inhibitor, that may be related to their larger neutral amino acid uptake and storage capability. This characteristic may allow memory NK cells to preserve their distinct functional features in the context of amino acid-restrictive conditions, such as tumor microenvironment (68).

Our results suggest that CD98 may contribute through mTORC1-dependent and -independent mechanisms to sustain CD16-triggered IFN-γ production, as direct interference with mTORC1 activation more modestly affects CD16-triggered IFN-γ expression than inhibition of antiporter function. In this regard, CD98-dependent, mTORC1-independent, sustained activation of c-Myc was shown to upregulate glycolytic and oxidative pathways and IFN-γ expression in murine NK cells (35). Finally, CD98 antiporter activity may also provide the necessary building blocks for IFN-γ protein synthesis.

On the other hand, the lower expression of CD71 transferrin receptor on CD57^+^ memory and conventional NK cells is in line with the progressive decrease of proliferative potential during NK differentiation (28); CD71 was reported to play an important role in the MCMV-driven adaptive NK cell expansion and *in vivo* protection (66); similarly, iron deficiency was shown to impair NK cell activation, metabolism, and antiviral function *in vivo* (69).

In conclusion, our analysis reveals that human FcεRIγ^-^ memory NK cells display a characteristic nutrient transporter expression pattern, that may support their enhanced *in vivo* persistence and CD16-dependent hyperresponsiveness. Our results also demonstrate that CD16 engagement is capable of rewiring nutrient transporter profile on both memory and conventional NK cells, in a mTORC1-dependent manner, and provide first evidence of the involvement of CD98 neutral amino acid antiporter in sustaining CD16-dependent cytokine response. The implications of this study contribute to a deeper comprehension of the metabolic equipment that supports NK cell functional capabilities, thus paving the way to the elaboration of immunometabolic strategies aimed at modulating NK cell responsiveness.

## Data Availability

The raw data supporting the conclusions of this article will be made available by the authors, without undue reservation.
