# Supplementary figures and images for: Nutrient transporter pattern in CD56dim NK cells: CD16 (FcγRIIIA)-dependent modulation and association with memory NK cell functional profile
Source: Front Immunol. 2024 Nov 13;15:1477776. doi: 10.3389/fimmu.2024.1477776 (PMC11599182; doi:10.3389/fimmu.2024.1477776)

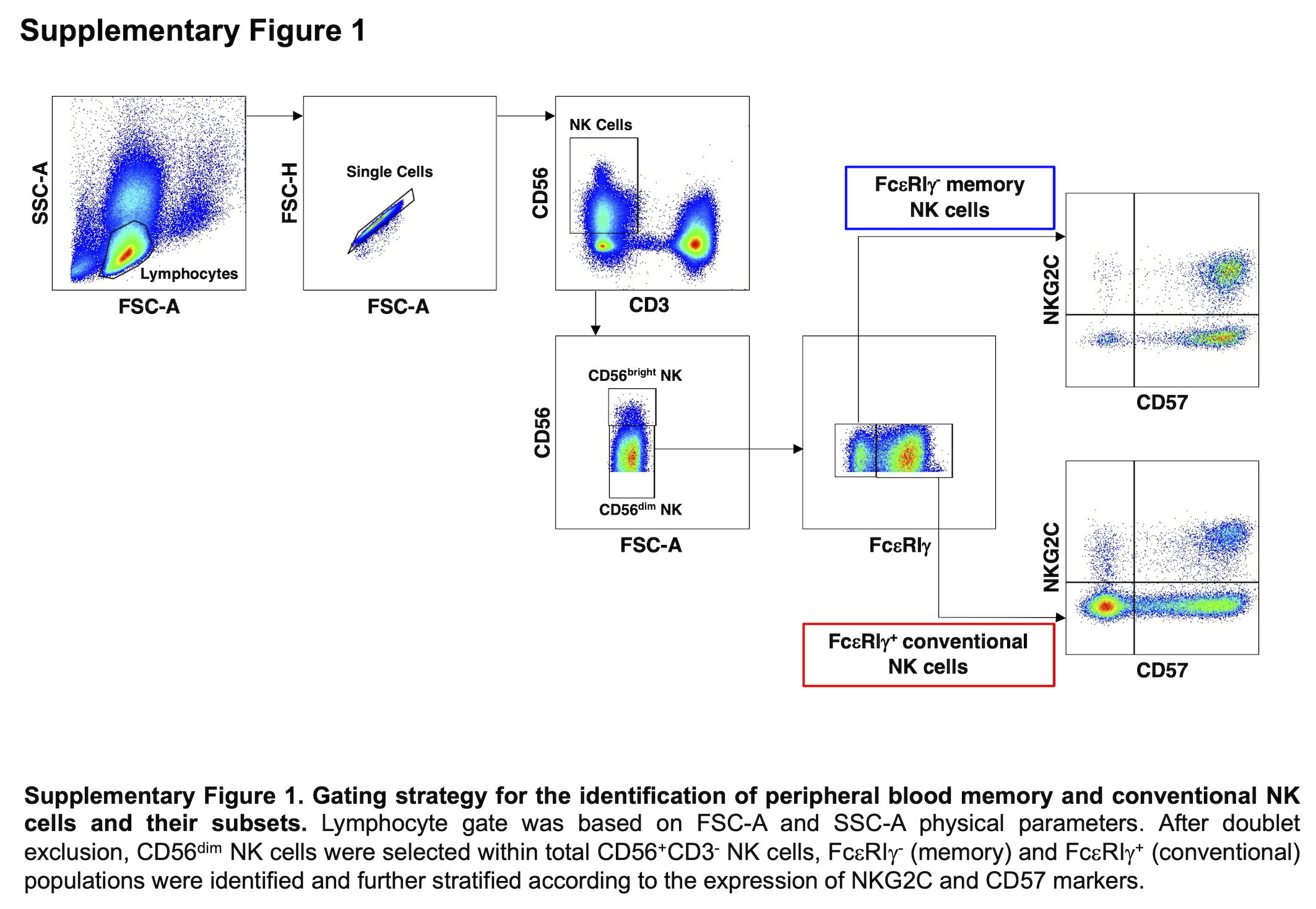

Supplement: Supplementary file 1 [file Image1.tiff]

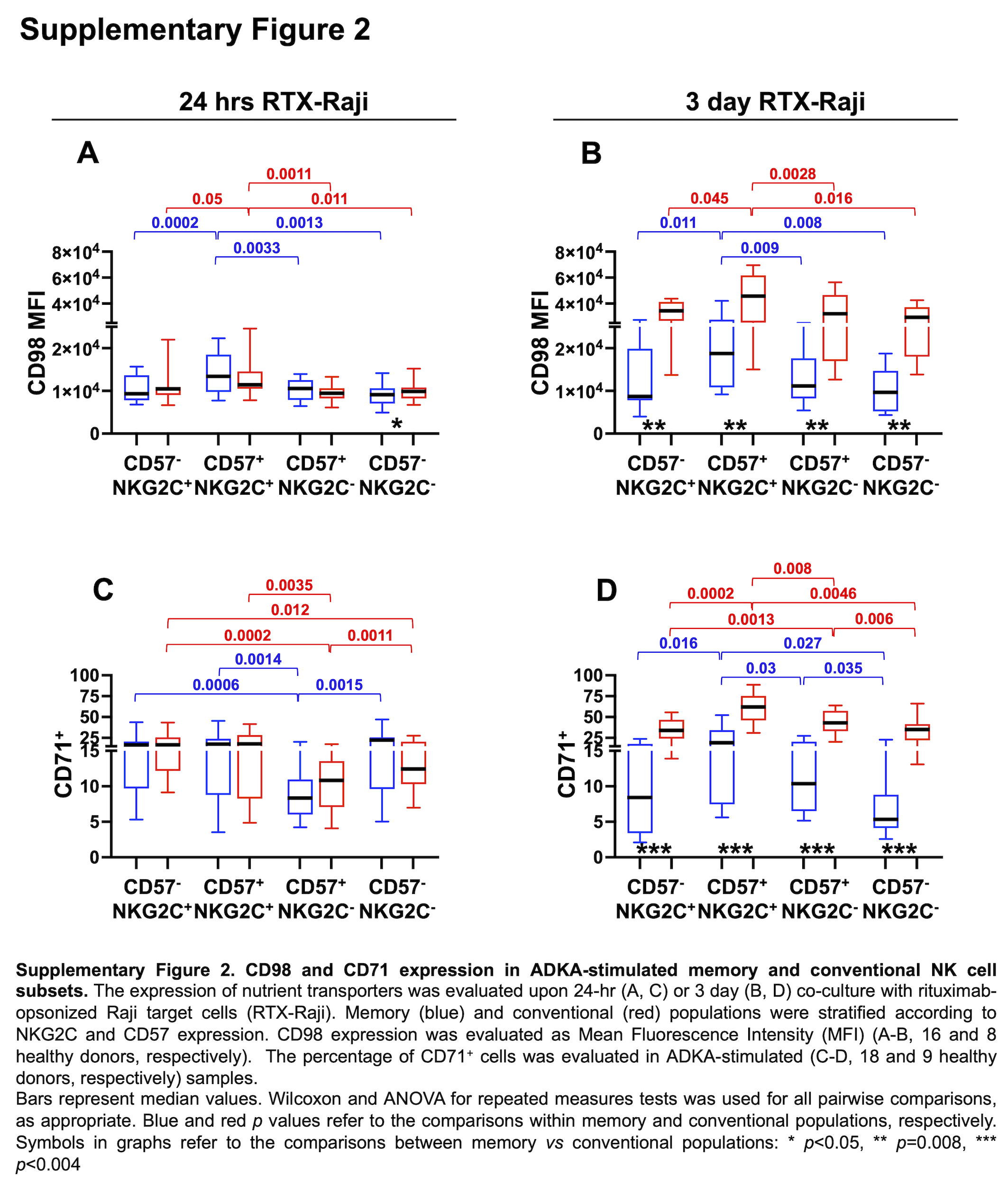

Supplement: Supplementary file 2 [file Image2.tiff]
